# The Mortality Burden of Multidrug-resistant Pathogens in India: A Retrospective, Observational Study

**DOI:** 10.1093/cid/ciy955

**Published:** 2018-11-08

**Authors:** Sumanth Gandra, Katie K Tseng, Anita Arora, Bhaskar Bhowmik, Matthew L Robinson, Bishnu Panigrahi, Ramanan Laxminarayan, Eili Y Klein

**Affiliations:** 1 Center for Disease Dynamics, Economics & Policy, Washington, DC; 2 Fortis Healthcare Ltd., Gurgaon, Haryana, India; 3 Division of Infectious Diseases, Center for Clinical Global Health Education, Johns Hopkins University School of Medicine, Baltimore, Maryland; 4 Princeton Environmental Institute, Princeton University, New Jersey; 5 Department of Global Health, University of Washington, Seattle; 6 Department of Emergency Medicine, Johns Hopkins School of Medicine; 7 Department of Epidemiology, Johns Hopkins Bloomberg School of Public Health, Baltimore, Maryland

**Keywords:** multidrug-resistant organisms, low- and middle-income countries, antimicrobial resistance, health care–acquired infections

## Abstract

**Background:**

The threat posed by antibiotic resistance is of increasing concern in low- and middle-income countries (LMICs) as their rates of antibiotic use increase. However, an understanding of the burden of resistance is lacking in LMICs, particularly for multidrug-resistant (MDR) pathogens.

**Methods:**

We conducted a retrospective, 10-hospital study of the relationship between MDR pathogens and mortality in India. Patient-level antimicrobial susceptibility test (AST) results for *Enterococcus* spp., *Escherichia coli*, *Staphylococcus aureus*, *Klebsiella pneumoniae*, *Acinetobacter baumannii*, *Pseudomonas aeruginosa*, and *Enterobacter* spp. were analyzed for their association with patient mortality outcomes.

**Results:**

We analyzed data on 5103 AST results from 10 hospitals. The overall mortality rate of patients was 13.1% (n = 581), and there was a significant relationship between MDR and mortality. Infections with MDR and extensively drug resistant (XDR) *E. coli*, XDR *K. pneumoniae*, and MDR *A. baumannii* were associated with 2–3 times higher mortality. Mortality due to methicillin-resistant *S. aureus* (MRSA) was significantly higher than susceptible strains when the MRSA isolate was resistant to aminoglycosides.

**Conclusions:**

This is one of the largest studies undertaken in an LMIC to measure the burden of antibiotic resistance. We found that MDR bacterial infections pose a significant risk to patients. While consistent with prior studies, the variations in drug resistance and associated mortality outcomes by pathogen are different from those observed in high-income countries and provide a baseline for studies in other LMICs. Future research should aim to elucidate the burden of resistance and the differential transmission mechanisms that drive this public health crisis.

## Teaser

There is a lack of understanding of the burden of antibiotic resistance in low- and middle-income countries. Using data from 10 hospitals across India, we found that the risk of mortality was significantly higher for patients with multidrug-resistant infections.

Antibiotic-resistant infections, particularly those caused by multidrug-resistant (MDR) organisms (MDROs), pose a major threat to global public health. Critically ill patients with prior antimicrobial exposure [1, 2] or comorbidities [2] are particularly vulnerable to infection with MDROs, which can increase mortality, hospitalization costs, and length of hospital stays [3–5]. Though antibiotic resistance negatively impacts patients globally [6], analyses of the burden of resistance have been understudied in low- and middle-income countries (LMICs) [4, 7–9], even though rising incomes, lower drug costs, and unregulated sales have led to increasing antibiotic use and higher rates of resistance [10–12].

While MDROs are a significant global concern, they pose an increased risk in LMICs, where a large proportion of health-care facilities have inadequate hospital environmental conditions and insufficient availability of standard infection prevention and control items [13, 14]. Improved awareness of the burden of antibiotic resistance can help lower resistance-related morbidity and mortality and is necessary for developing and marshalling support for interventions. However, surveillance in most LMICs is fragmented, at best. Where rates of resistance have been estimated, they are often substantially higher than in high-income countries (HICs) [4, 15], and studies of the burden of resistance have also shown relatively higher rates of resistance-related mortality [8, 9, 15]. To date, most studies of the burden of resistance in LMICs have been limited to single-center studies of modest sample size [4, 8] and restricted to intensive care units (ICUs), preventing generalizability to the larger patient population.

India is one of the largest LMICs and the largest consumer of antibiotics [12]. Widespread use of broad-spectrum agents has driven the spread of MDROs in both community and hospital settings [8, 9, 12, 13, 15]. Despite rapid increases in resistance and widespread acknowledgement of the issue, the mortality burden of antibiotic resistance remains largely understudied in India. We used multi-institutional hospital data from a large Indian hospital system to examine factors associated with mortality among patients tested for those MDROs that have been prioritized by the World Health Organization (WHO). The resulting data on the mortality burden of antibiotic resistance can aid in the development of policy efforts to prioritize antibiotic resistance as a public health threat in LMICs, as well as provide a baseline for future efforts to quantify the burden of resistance across LMICs.

## METHODS

### Study Design and Data Collection

We conducted a retrospective, multi-institutional, observational study across India using data from Fortis Healthcare Limited, an integrated health-care service provider. Antimicrobial susceptibility test (AST) results from January 2015 to December 2015 were collected from 10 tertiary and quaternary referral hospitals’ microbiology databases. The hospitals ranged in size from 120 to 350 beds and were geographically dispersed: 5 were in Northern India (4 in New Delhi district and 1 in Jaipur, Rajasthan), 2 in Western India (Mumbai, Maharashtra), 2 in Southern India (Bengaluru, Karnataka), and 1 in Eastern India (Kolkata, West Bengal). All hospitals were equipped with their own microbiology laboratories. There were 7 hospitals that used the VITEK 2 system (bioMérieux, Marcy l’Etoile, France) to conduct organism identification and AST; 3 hospitals used biochemical tests for organism identification and the Kirby-Bauer disk diffusion method for AST. All hospitals categorized AST results based on the Clinical and Laboratory Standards Institute criteria at the time of testing, except for colistin resistance to Enterobacteriaceae, in which results obtained from VITEK 2 (with agar dilution as the reference method) were interpreted based on the European Committee on Antimicrobial Susceptibility Testing guidelines.

Data obtained were patient-level AST results and mortality outcomes from hospital inpatient encounters. Mortality data were restricted to in-hospital mortality for the specific encounter. For patients with multiple isolates of a single organism from multiple specimen sources, we included only 1, giving preferential inclusion to isolates from blood or cerebrospinal fluid (CSF), followed by isolates from the lower respiratory system, wounds, urine, and any other source (eg, eye, gastrointestinal, genitourinary, upper respiratory, sterile fluid [non-CSF], stool, and tissue/biopsy). Additional demographic and clinical data included mortality outcome, age, sex, specimen source, location in the hospital (ie, non-ICU vs ICU), and place of infection acquisition (ie, community vs hospital). Isolates were considered as community-acquired infections if they were collected within 2 days of admission; otherwise, they were categorized as hospital-acquired infections.

### ESKAPE Pathogens

We examined resistance patterns for the common drugs used to treat the ESKAPE pathogens (*Enterococcus* spp., *Staphylococcus aureus*, *Klebsiella pneumoniae*, *Acinetobacter baumannii*, *Pseudomonas aeruginosa*, and *Enterobacter* spp.), listed as priority antibiotic-resistant pathogens by the WHO [16] (Supplementary Table 1). We also examined resistance patterns for *Escherichia coli*, due to its ubiquity. Pathogens were classified as either MDR or extensively drug resistant (XDR), based on drug-pathogen combinations proposed by the European Centre for Disease Prevention and Control and the US Centers for Disease Control and Prevention (Supplementary Tables 1 and 2) [17]. Methicillin-resistant *S. aureus* (MRSA) isolates were considered MDR due to the production of beta-lactamase enzymes, which typically confer resistance to beta-lactam antibiotics, including cephalosporins, carbapenems, and fluoroquinolones. We further classified MRSA isolates resistant to aminoglycosides, linezolid, tigecycline, or vancomycin as XDR. *Enterococcus* spp. were considered MDR if they were non-susceptible to vancomycin or teicoplanin. For Gram-negative organisms, only isolates tested against at least 1 agent in 3 or more antimicrobial classes were included in analyses. *E. coli* and *K. pneumoniae* were categorized by the following MDR categories: (1) non-susceptibility to 3 or more antimicrobial classes; (2) non-susceptibility to beta lactam/beta-lactamase inhibitors; and (3) non-susceptibility to all 5 antimicrobial classes, which we defined as XDR. Finally, for *P. aeruginosa* and *A. baumannii*, we defined MDR as non-susceptibility to 3 or more antimicrobial classes and XDR as non-susceptibility to all 5 antimicrobial classes.

### Statistical Analysis

To evaluate patient mortality in relation to MDR, we conducted multivariate logit regression analyses, adjusting for age, sex, hospital location, and specimen source. For overall analyses, patients with multiple organisms were collapsed to a single row and the highest resistance level was used. Sub-analyses were conducted for each pathogen, as well as groups of pathogens (ie, Gram-negative and Gram-positive), and were restricted based on the clinical significance of specific specimen sources and the availability of mortality data. *Enterococcus* infections were restricted to bloodstream, CSF, and urinary infections, based upon previous studies demonstrating significant clinical outcomes among patients with bacteriuria of *Enterococcus* spp. [18]. Because the 2 most common *Enterococcus* spp.—*Enterococcus faecalis* and *Enterococcus faecium*—can have distinct antimicrobial susceptibility profiles, we analyzed each species separately. Regression models were clustered at the hospital level to account for differences between hospitals in management and treatment of infections. All analyses were performed using Stata 14 (StataCorp, College Station, TX).

## RESULTS

**Figure 1.**
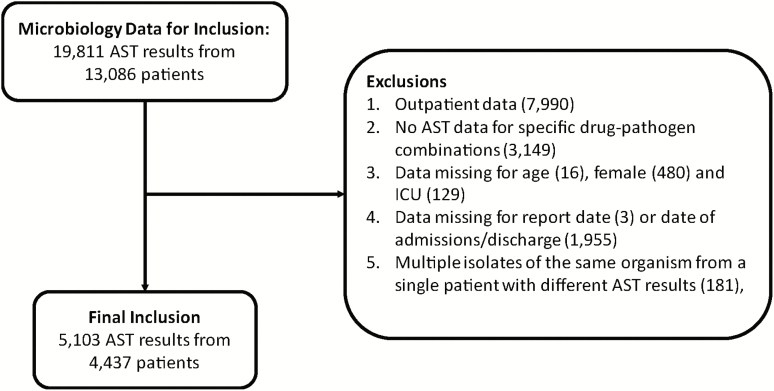
Study inclusion/exclusion flowchart of AST results. Abbreviations: AST, antimicrobial susceptibility testing; ICU, intensive care unit.

A total of 19811 AST results from 13086 patients were obtained from 10 hospitals between January and December 2015. Of these, 5103 records met all inclusion criteria (Figure 1). The overall mortality rate was 13.1% (n = 581) (Table 1); however, the mortality rate was higher in patients infected with *A. baumannii* (29.0%) and lower in patients infected with *E. coli* (8.8%) and *Staphylococcus aureus* (11.0%; Supplementary Table 2).

**Table 1.** Demographic and Clinical Characteristics of Patients With Culture-confirmed Bacterial Infections

	All Cases	Discharged Patients	Nonsurvivors
	N (%)^a^	n (%)^a^	n (%)^a^
**Total N**	4437 (100.0)	3856 (86.9)	581 (13.1)
Median age (IQR)	58 (40–69)	57 (38–69)	61 (48–70)
Age in years			
0–11	253 (5.7)	229 (5.9)	24 (4.1)
12–44	1070 (24.1)	970 (25.2)	100 (17.2)
45–64	1560 (35.2)	1334 (34.6)	226 (38.9)
>64	1554 (35.0)	1323 (34.3)	231 (39.8)
Female	1767 (39.8)	1561 (40.5)	206 (35.5)
ICU	1154 (26.0)	891 (23.1)	263 (45.3)
Organism^b^			
*Staphylococcus aureus*	282 (5.5)	251 (5.8)	31 (4.1)
*Enterococcus* spp.^c^	300 (5.9)	262 (6.0)	38 (5.1)
*Escherichia coli*	1907 (37.4)	1739 (40.0)	168 (22.3)
*Klebsiella pneumoniae*	1370 (26.9)	1118 (25.7)	252 (33.5)
*Enterobacter* spp.^d^	133 (2.6)	116 (2.7)	17 (2.3)
*Pseudomonas aeruginosa*	591 (11.6)	496 (11.4)	95 (12.6)
*Acinetobacter baumannii*	520 (10.2)	369 (8.5)	151 (20.1)

Abbreviations: ICU, intensive care unit; IQR, interquartile range.

^a^Unless otherwise indicated.

^b^For species-level analyses, the pathogen counts (N = 5103) include all isolates meeting multidrug-resistant testing criteria, including isolates obtained from the same patient for different organisms (ie, coinfecting pathogens).

^c^ *Enterococcus* spp. include *E. faecalis* (47.3%), *E. faecium* (42.3%), *E. gallinarum* (0.7%), and unknown *Enterococcus* spp. (9.3%).

^d^ *Enterobacter* spp. include *E. aerogenes* (10.5%), *E. cloacae* (67.7%), *E. dissolvens* (6.8%), and unknown *Enterobacter* spp. (15.0%).

Patients that died were older, on average, and were more likely to have obtained their isolate in the ICU (Table 1). They were also more likely to have a *K. pneumoniae* or *A. baumannii* infection, compared to discharged patients. Overall, mortality rates among patients with MDR infections were highest among those caused by Gram-negative bacteria (17.7%), as opposed to those caused by Gram-positive bacteria (10.8%), particularly in the ICU, where 26.9% of patients with Gram-negative MDR infections died, compared to 16.0% of patients with Gram-positive MDR infections.

Controlling for age, sex, site of infection, and the number of coinfections, we found increased odds of mortality among patients with MDR infections (odds ratio [OR] 1.57, 95% confidence interval [CI] 1.14–2.16) and XDR infections (OR 2.65, 95% CI 1.81–3.88; Table 2). Restricting the analysis to non-ICU inpatients only, patients with MDR infections had significantly higher odds of mortality (OR 1.74, 95% CI 1.06–2.87), as did patients with XDR infections (OR 2.87, 95% CI 1.80–4.57). Similarly, ICU patients had a higher likelihood of mortality if they had an XDR infection (OR 2.07, 95% CI 1.24–3.26). However, we found these associations were largely driven by patients with Gram-negative MDR infections, where the odds of mortality for XDR infections were 3.15 (95% CI 2.01–4.94) in the non-ICU and 2.01 (95% CI 1.12–3.59) in the ICU (Supplementary Table 3). In contrast, XDR Gram-positive infections were only significantly associated with mortality among non-ICU inpatients (OR 2.93, 95% CI 1.03–8.37). The infection-onset location (community vs hospital) was not a significant factor for any organism (data not shown).

**Table 2.** Mortality Odds Among Patients With Culture-confirmed Bacterial Infections

	All Cases (N = 4437)	Inpatient (Non-ICU) (n = 3282)	ICU (n = 1155)
	OR (95% CI)	OR (95% CI)	OR (95% CI)
Resistance pattern			
Non-MDR	Referent	Referent	Referent
MDR^a^	1.57 (1.14–2.16)**	1.74 (1.06–2.87)*	1.24 (0.81–1.88)
XDR^b^	2.65 (1.81–3.88)***	2.87 (1.80–4.57)***	2.01 (1.24–3.26)**
Age (years)			
0–11	Referent	Referent	Referent
12–44	1.09 (0.68–1.75)	1.14 (0.65–1.99)	1.05 (0.46–2.41)
45–64	1.60 (0.88–2.90)	1.52 (0.84–2.75)	1.82 (0.70–4.71)
>64	1.62 (0.81–3.28)	1.59 (0.81–3.13)	1.74 (0.58–5.24)
Female	0.97 (0.79–1.18)	0.80 (0.61–1.04)	1.34 (1.11–1.61)**
Site of infection			
Other	2.39 (1.52–3.74)***	2.34 (1.61–3.38)***	2.67 (1.20–5.95)*
Urine	Referent	Referent	Referent
Wound	1.26 (0.96–1.65)	1.20 (0.74–1.94)	1.38 (0.62–3.11)
Lower respiratory	3.45 (2.19–5.42)***	2.45 (1.28–4.69)**	3.75 (2.49–5.64)***
Blood/CSF	5.34 (2.58–11.08)***	5.29 (2.30–12.17)***	4.27 (3.00–6.09)***
Coinfection^c^			
None	Referent	Referent	Referent
Single	1.34 (1.02–1.76)*	1.18 (0.82–1.69)	1.51 (0.82–2.78)
Multiple	1.54 (0.96–2.47)	1.69 (1.22–2.35)**	1.48 (0.50–4.36)

Logit regression is with clustered standard errors at the hospital level.

Abbreviations: CI, confidence interval; CSF, cerebrospinal fluid; ICU, intensive care unit; MDR, multidrug-resistant; OR, odds ratio; XDR, extensively drug resistant.

^a^MDR is defined as nonsusceptibility to 1 or more agents in 3 or more antimicrobial classes (ie, aminoglycosides, third-/fourth-generation cephalosporins, fluoroquinolones, beta-lactam/beta-lactamase inhibitors, and carbapenems) for Gram-negative organisms; nonsusceptibility to oxacillin and/or cefoxitin (anti-staphylococcal beta-lactams) for Gram-positive *Staphylococcus aureus*; and non-susceptibility to vancomycin and/or teicoplanin (glycopeptides) for Gram-positive *Enterococcus* spp.

^b^XDR is defined as nonsusceptibility to 1 or more agents in all 5 antimicrobial classes for Gram-negative organisms; and nonsusceptibility to oxacillin and/or cefoxitin and to 1 or more agents in the antimicrobial class aminoglycosides for Gram-positive *S. aureus*.

^c^Isolation of 2 or more pathogens (ie, *S. aureus*, *Enterococcus* spp., *Escherichia coli*, *Klebsiella pneumoniae*, *Enterobacter* spp., *Pseudomonas aeruginosa*, and *Acinetobacter baumannii*) from a single patient.

*P < .05. **P < .01. ***P < .001.

### Gram-negative Infections: *Escherichia coli*, *Klebsiella pneumoniae, Pseudomonas aeruginosa,* and *Acinetobacter baumannii*

Patients with *E. coli* infections resistant to multiple drug classes, including beta-lactam/beta-lactamase inhibitors, had higher odds of mortality after controlling for other factors, though for *K. pneumoniae,* only XDR infections were significantly associated with higher mortality (Table 3). Compared to non-MDR *E. coli*, the odds of mortality were 2.63 (95% CI 1.29–5.35) times higher for MDR *E. coli*; 2.23 (95% CI 1.65–3.01) times higher for beta-lactam/beta-lactamase inhibitor resistant *E. coli*; and 2.34 (95% CI 1.40–3.90) times higher for XDR *E. coli*. Compared to non-MDR *K. pneumoniae,* the odds of mortality were 2.29 (95% CI 1.45–3.62) times higher for XDR *K. pneumoniae.* Among patients tested for colistin-resistance, 9 (0.8%) *E. coli* cases and 38 (4.6%) *K. pneumoniae* cases were non-susceptible, with 2 and 10 deaths reported among them, respectively.

**Table 3.** Mortality Odds Among Patients With Gram-negative Infections

	*Escherichia coli* (n = 1907)	*Klebsiella pneumoniae* (n = 1370)	*Pseudomonas aeruginosa* (n = 591)	*Acinetobacter baumannii* (n = 520)
	OR (95% CI)	OR (95% CI)	OR (95% CI)	OR (95% CI)
Resistance pattern				
Non-MDR	Referent	Referent	Referent	Referent
MDR^a^	2.63 (1.29–5.35)**	1.47 (0.52–4.11)	1.15 (0.65–2.04)	2.81 (1.50–5.27)**
MDR + beta-lactamase inhibitors^b^	2.23 (1.65–3.01)***	1.20 (0.61–2.37)	NA	NA
XDR^c^	2.34 (1.40–3.90)**	2.29 (1.45–3.62)***	1.76 (0.84–3.72)	2.26 (0.77–6.61)
Age (years)				
0–11	Referent	Referent	Referent	Referent
12–44	0.94 (0.65–1.36)	1.80 (0.90–3.62)	0.64 (0.17–2.38)	0.83 (0.30–2.27)
45–64	1.50 (0.65–3.46)	3.05 (1.38–6.74)**	0.83 (0.21–3.22)	1.09 (0.45–2.68)
>64	1.19 (0.42–3.39)	3.20 (1.23–8.31)*	0.74 (0.24–2.22)	1.97 (0.63–6.14)
Female	0.83 (0.54–1.27)	0.84 (0.60–1.17)	1.05 (0.73–1.50)	1.40 (0.99–1.97)
ICU	1.57 (0.89–2.79)	2.10 (1.36–3.25)**	2.09 (1.25–3.49)**	1.66 (1.28–2.16)***
Site of infection				
Other	1.62 (1.02–2.57)*	2.47 (1.01–6.07)*	1.86 (0.91–3.83)	2.69 (0.62–11.63)
Urine	Referent	Referent	Referent	Referent
Wound	0.61 (0.32–1.14)	1.45 (0.83–2.55)	1.33 (0.68–2.59)	2.09 (0.30–14.54)
Lower respiratory	1.61 (1.05–2.47)*	2.70 (1.52–4.79)**	2.67 (1.77–4.03)***	3.77 (1.58–8.99)**
Blood/CSF	3.28 (2.21–4.85)***	6.67 (2.32–19.15)***	2.76 (1.24–6.13)*	4.52 (1.41–14.46)*
Coinfection^d^				
None	Referent	Referent	Referent	Referent
Single	2.41 (1.65–3.52)***	1.55 (1.28–1.88)***	2.16 (1.38–3.38)**	1.35 (0.70–2.60)
Multiple	3.92 (2.15–7.13)***	2.31 (1.37–3.89)**	2.96 (1.62–5.41)***	2.20 (1.38–3.50)**

Logit regression is with clustered standard errors at the hospital level.

Abbreviations: CI, confidence interval; CSF, cerebrospinal fluid; ICU, intensive care unit; MDR, multidrug-resistant; NA, not applicable; OR, odds ratio; XDR, extensively drug resistant.

^a^Nonsusceptibility to 1 or more agents in 3 or more antimicrobial classes (ie, aminoglycosides, third-/fourth-generation cephalosporins, fluoroquinolones, beta-lactamase inhibitors, and carbapenems), excluding non-susceptibility to beta-lactamase inhibitors.

^b^Nonsusceptibility to 1 or more agents in the antimicrobial class of beta-lactamase inhibitors.

^c^Nonsusceptibility to 1 or more agents in all 5 aforementioned antimicrobial classes.

^d^Isolation of 2 or more pathogens (ie, *Staphylococcus aureus*, *Enterococcus* spp., *E. coli*, *K. pneumoniae*, *Enterobacter* spp., *P. aeruginosa*, and *A. baumannii*) from a single patient.

*P < .05. **P < .01. ***P < .001.

Among patients with *P. aeruginosa* infections, mortality was not significantly associated with MDR infections (OR 1.15, 95% CI 0.65–2.04) or XDR infections (OR 1.76, 95% CI 0.84–3.72). However, among patients with *A. baumannii* infections, MDR was associated with 2.81 (95% CI 1.50–5.27) times higher odds of mortality than similar susceptible infections (Table 3). Notably, patients with *A. baumannii* infections also had higher likelihoods of dying if they were located in the ICU (OR 1.66, 95% CI 1.28–2.16). *A. baumannii* and *P. aeruginosa* infections of the lower-respiratory system were also strongly associated with a greater likelihood of mortality.

### Gram-positive Infections: *Staphylococcus aureus* and *Enterococcus* spp

Infections with more than 1 pathogen (ie, coinfections) comprised a small proportion of all Gram-positive infections and were not found to be significantly associated with either *S. aureus* or *Enterococcus* spp. infections. Therefore, we restricted results of all patients with Gram-positive infections to those without coinfections. Controlling for the higher mortality rates associated with *S. aureus* bacteremia or CSF infections, no significant difference in mortality rates was observed between patients with MRSA infections compared to those with methicillin-susceptible *S. aureus* (MSSA) infections (Table 4). However, patients with MRSA infections that were also non-susceptible to aminoglycosides had a greater likelihood of mortality, compared to MSSA-infected patients (OR 2.75, 95% CI 1.16–6.52). Among patients who acquired MRSA infections with an additional resistance to linezolid (n = 1) or a reduced susceptibility (ie, intermediate resistance) to vancomycin (n = 1) or teicoplanin (n = 1), all survived.

**Table 4.** Mortality Odds Among Patients With Gram-positive Infections

	*Staphylococcus aureus* (n = 237)	*Enterococcus* spp. (n = 192)	*Enterococcus faecalis* (n = 99)	*Enterococcus faecium* (n = 40)
	OR (95% CI)	OR (95% CI)	OR (95% CI)	OR (95% CI)
Resistance pattern				
MSSA or glycopeptide sensitive	Referent	NA	Referent	Referent
MRSA^a^	0.17 (0.01–2.15)	NA	NA	NA
MRSA + aminoglycosides^b^	2.75 (1.16–6.52)*	NA	NA	NA
Glycopeptide resistant^c^	NA	1.09 (0.36–3.33)	2.55 (0.13–51.65)	0.98 (0.08–11.58)
Age (years)				
0–11	Referent	Referent	–	–
12–44	1.09 (0.23–5.22)	4.29 (0.75–24.47)	Referent	Referent
45–64	0.47 (0.09–2.62)	7.74 (0.68–88.45)	1.69 (0.04–75.57)	2.88 (0.35–23.89)
>64	0.65 (0.12–3.64)	0.70 (0.12–4.15)	0.07 (0.00–1.37)	
Female	0.47 (0.20–1.11)	0.93 (0.29–2.96)	0.49 (0.12–2.04)	1.34 (0.12–15.07)
ICU	1.45 (0.37–5.58)	1.59 (0.62–4.10)	4.78 (2.52–9.06)***	0.45 (0.04–4.72)
Site of infection				
Urine	NA	Referent	Referent	Referent
Wound	Referent	–	–	–
Lower respiratory	3.81 (1.29–11.30)*	–	–	–
Blood/CSF	4.56 (1.61–12.94)**	7.13 (0.61–83.48)	9.57 (0.49–187.79)	1.35 (0.06–31.27)

Logit regression is with clustered standard errors at the hospital level. – denotes observations excluded from analysis due to clinical insignificance or no observed mortalities. Patients with coinfections were excluded from analysis due to small sample sizes and statistical insignificance in relation to mortality. *Enterococcus* spp. includes the species *E. avium, E. faecalis, E. faecium, E. gallinarum,* and unknown *Enterococcus* spp.

Abbreviations: CI, confidence interval; CSF, cerebrospinal fluid; ICU, intensive care unit; MSSA, methicillin-susceptible *S. aureus*; MRSA, methicillin-resistant *S. aureus*; NA, not applicable; OR, odds ratio.

^a^Nonsusceptibility to oxacillin and/or cefoxitin (anti-staphylococcal beta-lactams), excluding nonsusceptibility to aminoglycosides; no deaths occurred among MRSA infections with additional resistance to linezolid.

^b^Nonsusceptibility to oxacillin and/or cefoxitin and to 1 or more agents in the antimicrobial class of aminoglycosides.

^c^Nonsusceptibility to vancomycin and/or teicoplanin (glycopeptides).

*P < .05. **P < .01. ***P < .001.

Glycopeptide resistance in *Enterococcus* spp. was not associated with an increased likelihood of mortality, regardless of species, after controlling for patient demographics (Table 4). Non-susceptibility to linezolid was detected in 2 (4.8%) patients with a glycopeptide-resistant *Enterococcus* infection, 1 of whom died.

## DISCUSSION

As rates of antibiotic use in LMICs converge with those of HICs [12], there has been increasing acknowledgment of and concern over the problem of antibiotic resistance in LMICs. However, current knowledge of the burden of antibiotic resistance in LMICs, particularly related to MDR infections, is severely lacking. In this study, we used multi-institutional antimicrobial-susceptibility data to assess the burden of MDR among the WHO’s priority list of antimicrobial resistance (AMR) bacteria on patient mortality in India. While India is the largest global antibiotic consumer, on a per-capita basis it has antibiotic consumption levels similar to other LMICs [12].

Our results indicate that patients who acquire MDR bacterial infections, as opposed to similar drug-susceptible infections, have greater odds of mortality. Interestingly, we observed higher odds of mortality among patients with MDR and XDR infections whose isolates were obtained outside the ICU. These results may be due to differences in severity of illnesses, which we were unable to control for. Additionally, we only examined the first isolate from an individual, and thus the future course of hospitalization (which may have included the ICU) was not taken into account.

Given the high rates of hospital infections caused by MDR Gram-negative organisms in LMICs [19, 20], we examined their relative mortality impact, compared to Gram-positive infections. We found that Gram-negative MDRO infections were associated with higher mortality rates, especially among patients in the ICU. However, the high odds of mortality associated with Gram-negative XDR infections among all patients suggests that more rapid identification of Gram-negative infections in both ICU and non-ICU patients, particularly those with bacteremia or lower-respiratory infections, may help reduce the clinical burden of MDR and improve mortality outcomes overall.

In India, infections with MDR and XDR Gram-negative bacteria are frequent [8, 13, 21, 22] and pose a significant challenge to clinicians due to severely limited therapeutic options. Once a pillar for empiric antibiotic therapy, third-generation cephalosporins are largely ineffective against infections of extended spectrum beta-lactamase–producing *Enterobacteriaceae* [13], and rates of carbapenem resistance are as high as 57% in some Indian health-care settings [21]. High rates of resistance are strongly related to patient outcomes, as indicated by our study; patients infected with MDR *E. coli,* XDR *K. pneumoniae*, and MDR *A. baumannii* were 2 to 3 times more likely to die than patients with non-MDR infections. These associated clinical outcomes appear consistent with existing research in smaller studies, showing MDR and XDR to be predictors of worse clinical outcomes, particularly among patients with XDR *K. pneumoniae* bacteremia, which in LMICs have been attributed to mortality rates up to 32–50% [4, 9].

Our findings also highlight the clinical importance of MDR and XDR strains of *A. baumannii* infections. The increased odds of mortality associated with MDR *A. baumannii* infections were consistent with previous studies linking carbapenem resistance to higher mortality rates and longer hospitalizations [3]. Able to survive in the hospital environment for extended periods of time [23], *A. baumannii* and *P. aeruginosa* are commonly implicated in device-associated infections. In particular, *A. baumannii* has a remarkable propensity for acquiring genetic material from other organisms, allowing it to develop extensive resistance over the last few decades [23].

For *S. aureus* infections, we found a significant difference in mortality between MRSA infections, with additional resistance to aminoglycosides and MSSA infections. These findings support existing evidence that MRSA infections are more likely to be resistant to other antibiotics than MSSA infections [24, 25] and suggest that MDR may, in part, be driving higher inpatient mortality rates among *S. aureus* infections, as demonstrated in previous studies [26].

Since the late 1970s, *Enterococcus* spp. have been recognized as a leading cause of health care–associated urinary and blood-stream infections [27]. However, contrary to findings from a 2016 systematic review [28], which reported an increased unadjusted mortality risk associated with vancomycin-resistant enterococci infections, our study found no significant impact of glycopeptide resistance in *Enterococcus* infections. This result held true after restricting the analysis to blood cultures only (data not shown), which may be explained by the relatively few deaths associated with *Enterococcus* spp. in our study (35, 12.1%), as well as the limited number of isolates of clinical significance.

There are several limitations to the study. The lack of complete clinical data precluded us from capturing potentially important variables, including severity of illness, comorbid conditions, and time to effective therapy, all of which are associated with mortality among MDR-infected patients [29, 30]. However, previous studies controlling for comorbidities and severities of illnesses have shown independent associations between increased mortality and inappropriate antimicrobial therapy for those infections most commonly caused by MDROs [30, 31]. In addition, though we classified infections as hospital- or community-associated, based on the time of isolate collection, we lacked more detailed information on the timing of collection, as well as information on prior hospitalizations, which prevented us from a more accurate classification of infection-acquisition location. This may explain why we found no difference in mortality rates related to community vs hospital onset, even though prior studies have shown varied rates of resistance and attributable mortality rates based on infection-onset locations [9]. Alternatively, the high rates of MDRO in the community [8, 9] may mean that there is little difference between the pathogens transmitted in the hospital and the community. Further study is needed to understand the burden of high community rates of MDROs in resource-limited settings. Finally, while our study was based on multi-institutional data across India, our findings were not able to capture the heterogeneity of India’s health-care landscape and may not be generalizable to specific communities or clinical settings.

While data were observational, preventing establishment of causality, our results provide strong quantification of the association between mortality and MDR patterns in a representative LMIC, and highlight the significant threat MDR and XDR pathogens pose to human health in developing countries. The high mortality odds underscore the urgent need to improve understanding of the burden of mortality and morbidity attributable to MDR and XDR Gram-negative pathogens in LMICs. In fact, our results are likely an underestimate of the overall burden of MDR infections: we only examined mortality, but resistant infections are also associated with increases in morbidity and hospital costs [5]. Future research should prospectively enroll patients with MDR pathogens and adequate controls to improve understanding of the burden of resistance and to provide greater insight into the attributable risks of morbidity and mortality.

Research aimed at understanding the genetic and biochemical mechanisms of antimicrobial resistance in XDR Gram-negative pathogens is critically needed, as available therapeutic options, including those in the pipeline, are ineffective against existing molecular mechanisms, such as New Delhi metallobeta-lactamase, which are highly prevalent among XDR Gram-negative pathogens. Furthermore, increased surveillance is necessary to understand the extent of resistance in the community and the hospital, and to better quantify the impact that community transmission has on hospital infection patterns. As resistance can spread worldwide rapidly, investment by both LMICs and HICs into these research areas should be of utmost priority to combat the emergence and spread of MDR pathogens and conserve the global efficacy of antibiotics.

## Supplementary Data

Supplementary materials are available at *Clinical Infectious Diseases* online. Consisting of data provided by the authors to benefit the reader, the posted materials are not copyedited and are the sole responsibility of the authors, so questions or comments should be addressed to the corresponding author.

ciy955_suppl_Supplementary_Table

## Notes

***Financial support.*** This work was supported by the Bill & Melinda Gates Foundation to The Global Antimicrobial Resistance Repository (grant number OPP1112355 to E. Y. K., S. G., and K. K. T.) and the Global Antibiotic Resistance Partnership (grant number OPP1135911 to E. Y. K., S. G., and K. K. T.) at the Center for Disease Dynamics, Economics & Policy.

***Potential conflicts of interest.*** R. L. was supported by Centers for Disease Control (grant number 16IPA1609427) at Princeton University. M. L. R. was supported in part by an Antibacterial Resistance Leadership Group fellowship (National Institute of Allergy and Infectious Diseases grant number UM1AI104681). All other authors report no potential conflicts. All authors have submitted the ICMJE Form for Disclosure of Potential Conflicts of Interest. Conflicts that the editors consider relevant to the content of the manuscript have been disclosed.
