# Supplementary material for: The Mortality Burden of Multidrug-resistant Pathogens in India: A Retrospective, Observational Study
Source: Clin Infect Dis. 2018 Nov 8;69(4):563–70. doi: 10.1093/cid/ciy955 (PMC6669283; doi:10.1093/cid/ciy955)
Supplement: ciy955_suppl_Supplementary_Table [file ciy955_suppl_supplementary_table.docx]

| **Supplementary Table 1. Pathogen-antimicrobial combinations** | | |
| --- | --- | --- |
| Pathogen | Antimicrobial class | Antibiotic agent |
| Gram-positive bacteria | | |
| *Staphylococcus aureus* | Anti-staphylococcal beta-lactams | Oxacillin  Cefoxitin |
|  | Aminoglycosides | Gentamicin |
|  | Oxazolidinones | Linezolid |
|  | Glycylcyclines | Tigecycline |
|  | Glycopeptide | Vancomycin  Teicoplanin |
| *Enterococcus* spp.  *(E. faecalis, E. faecium)* | Glycopeptides | Vancomycin  Teicoplanin |
|  | Oxazolidinones | Linezolid |
| Gram-negative bacteria |  |  |
| *Escherichia coli*,  *Klebsiella pneumoniae*, *Enterobacter* spp. | Aminoglycosides | Gentamicin  Tobramycin  Amikacin  Netilmicin |
|  | 3rd generation cephalosporins | Cefotaxime  Ceftriaxone  Ceftazidime |
|  | Fluoroquinolones | Ciprofloxacin |
|  | Beta-lactam/beta-lactamase inhibitors | Ticarcillin-clavulanate  Piperacillin-tazobactam  Amoxicillin-clavulanate |
|  | Carbapenems | Imipenem  Meropenem  Doripenem  Ertapenem |
| *Pseudomonas aeruginosa*, *Acinetobacter baumannii* | Aminoglycosides | Gentamicin  Tobramycin  Amikacin  Netilmicin |
|  | Third/fourth-generation cephalosporins | Ceftazidime  Cefepime |
|  | Fluoroquinolones | Ciprofloxacin  Levofloxacin |
|  | Beta-lactam/beta-lactamase inhibitors | Ticarcillin-clavulanate  Piperacillin-tazobactam |
|  | Carbapenems | Imipenem  Meropenem  Doripenem |
|  | | |
|  | | |

| **Supplementary Table 2. Demographic and clinical characteristics of patients with culture-confirmed bacterial infections** | | | | | | | | |
| --- | --- | --- | --- | --- | --- | --- | --- | --- |
|  | All cases | *Staphylococcus aureus* | *Enterococcus* spp*.^a^* | *Escherichia coli* | *Klebsiella pneumoniae* | *Enterobacter* spp.^b^ | *Pseudomonas aeruginosa* | *Acinetobacter baumannii* |
|  | N (%)^c^ | n (%)^c^ | n (%)^c^ | n (%)^c^ | n (%)^c^ | n (%)^c^ | n (%)^c^ | n (%)^c^ |
| **Total N^d^** | 4,437 (100.0) | 282 (5.5) | 300 (5.9) | 1,907 (37.4) | 1,370 (26.9) | 133 (2.6) | 591 (11.6) | 520 (10.2) |
| Median age (IQR) | 58 (40-69) | 50 (28-63) | 61 (42-72) | 58 (38-70) | 60 (44-70) | 53 (30-64) | 59 (41-69) | 58 (40-68) |
| Age in years |  |  |  |  |  |  |  |  |
| 0-11 | 253 (5.7) | 38 (13.5) | 15 (5.0) | 104 (5.5) | 56 (4.1) | 8 (6.0) | 21 (3.6) | 30 (5.8) |
| 12-44 | 1,070 (24.1) | 79 (28.0) | 66 (22.0) | 484 (25.4) | 287 (21.0) | 42 (31.6) | 147 (24.9) | 117 (22.5) |
| 45-64 | 1,560 (35.2) | 102 (36.2) | 101 (33.7) | 635 (33.3) | 506 (36.9) | 52 (39.1) | 209 (35.4) | 189 (36.4) |
| >64 | 1,554 (35.0) | 63 (22.3) | 118 (39.3) | 684 (35.9) | 521 (38.0) | 31 (23.3) | 214 (36.2) | 184 (35.4) |
| Female | 1,767 (39.8) | 98 (34.8) | 138 (46.0) | 872 (45.7) | 494 (36.1) | 46 (34.6) | 179 (30.3) | 160 (30.8) |
| ICU | 1,154 (26.0) | 51 (18.1) | 75 (25.0) | 381 (20.0) | 453 (33.1) | 35 (26.3) | 179 (30.3) | 216 (41.5) |
| **Non-survivors** | 581 (13.1) | 31 (11.0) | 38 (12.7) | 168 (8.8) | 252 (18.4) | 17 (12.8) | 95 (16.1) | 151 (29.0) |
| Note: ICU = intensive care unit, IQR = interquartile range.  ^a^ *Enterococcus* spp. include *E. faecalis* (47.3%), *E. faecium* (42.3%), *E. gallinarum* (0.7%), and unknown *Enterococcus* spp. (9.3%).  ^b^ *Enterobacter* spp. include *E. aerogenes* (10.5%), *E. cloacae* (67.7%), *E. dissolvens* (6.8%), and unknown *Enterobacter* spp. (15.0%).  ^c^ Unless otherwise indicated.  ^d^ For species-level analyses, the pathogen counts include all isolates meeting MDR testing criteria, including isolates obtained from the same patient for different organisms (i.e., co-infecting pathogens). | | | | | | | | |

| **Supplementary Table 3. Mortality odds among inpatients with culture-confirmed bacterial infections** | | | | | |
| --- | --- | --- | --- | --- | --- |
|  | **Inpatient department (non-ICU)** | | **ICU** | | |
|  | Gram-positive  n = 432 | Gram-negative  n = 2,809 | | Gram-positive  n = 119 | Gram-negative  n = 1,029 |
|  | OR (95% CI) | OR (95% CI) | | OR (95% CI) | OR (95% CI) |
| Resistance pattern |  |  | |  |  |
| Non-MDR | Referent | Referent | | Referent | Referent |
| MDR^a^ | 1.03 (0.29-3.70) | 2.05 (1.22-3.45)** | | 0.52 (0.09-2.85) | 1.28 (0.76-2.16) |
| XDR^b^ | 2.93 (1.03-8.37)* | 3.15 (2.01-4.94)*** | | 1.84 (0.27-12.54) | 2.01 (1.12-3.59)* |
| Age (years) |  |  | |  |  |
| 0-11 | Referent | Referent | | Referent | Referent |
| 12-44 | 1.48 (0.23-9.37) | 1.07 (0.76-1.53) | | 0.67 (0.04-11.64) | 1.13 (0.52-2.45) |
| 45-64 | 1.19 (0.19-7.48) | 1.49 (1.00-2.22)* | | 1.84 (0.46-7.43) | 2.01 (0.72-5.63) |
| >64 | 0.70 (0.09-5.32) | 1.72 (0.97-3.05) | | 1.36 (0.17-11.16) | 1.94 (0.61-6.24) |
| Female | 0.60 (0.46-0.79)*** | 0.82 (0.59-1.12) | | 0.67 (0.19-2.37) | 1.39 (1.16-1.67)*** |
| Site of infection |  |  | |  |  |
| Other | NA | 2.55 (1.77-3.66)*** | | NA | 2.78 (1.24-6.20)* |
| Urine | Referent | Referent | | Referent | Referent |
| Wound | 0.65 (0.35-1.22) | 1.24 (0.66-2.33) | | 0.58 (0.05-6.87) | 1.60 (0.61-4.19) |
| Lower respiratory | 0.91 (0.29-2.88) | 2.63 (1.30-5.31)** | | 3.40 (0.91-12.66) | 3.84 (2.17-6.80)*** |
| Blood/CSF | 1.78 (0.79-3.99) | 5.81 (2.53-13.34)*** | | 5.85 (1.27-27.02)* | 4.14 (2.62-6.56)*** |
| Coinfection^c^ |  |  | |  |  |
| None | Referent | Referent | | Referent | Referent |
| Single | 1.50 (0.68-3.27) | 1.22 (0.85-1.74) | | 1.10 (0.32-3.81) | 1.56 (0.85-2.84) |
| Multiple | 2.45 (0.78-7.72) | 1.60 (1.08-2.35)* | | 1.29 (0.13-12.62) | 1.59 (0.52-4.81) |
| Note: Logit regression with clustered standard errors at the hospital level; CI = confidence interval, CSF = cerebrospinal fluid, ED = emergency department, ICU = intensive care unit, MDR = multi-drug resistant, NA = not applicable, OR = odds ratio, XDR = extensively-drug resistant  *p<0.05, **p<0.01, ***p<0.001  ^a^ MDR is defined as non-susceptibility to 1 or more agents in 3 or more antimicrobial classes (i.e., aminoglycosides, 3rd/4th generation cephalosporins, fluoroquinolones, beta-lactam/beta-lactamase inhibitors, and carbapenems) for Gram-negative organisms (*Escherichia coli*, *Klebsiella pneumoniae, Enterobacter* spp*., Pseudomonas aeruginosa,* and *Acinetobacter baumannii*); non-susceptibility to oxacillin and/or cefoxitin (anti-staphylococcal beta-lactams) for Gram-positive *Staphylococcus aureus*; and non-susceptibility to vancomycin and/or teicoplanin (glycopeptides) for Gram-positive *Enterococcus* spp*.*  ^b^ XDR is defined as non-susceptibility to 1 or more agents in all 5 antimicrobial classes for Gram-negative organisms; non-susceptibility to oxacillin and/or cefoxitin and to 1 or more agents in the antimicrobial class aminoglycosides for Gram-positive *S. aureus*.  ^c^ Isolation of 2 or more pathogens (i.e., *S. aureus*, *Enterococcus* spp., *E. coli*, *K. pneumoniae*, *Enterobacter* spp., *P. aeruginosa*, and *A. baumannii*) from a single patient. | | | | | |
